# An End-to-End Deep Neural Network for Autonomous Driving Designed for Embedded Automotive Platforms

**DOI:** 10.3390/s19092064

**Published:** 2019-05-03

**Authors:** Jelena Kocić, Nenad Jovičić, Vujo Drndarević

**Affiliations:** School of Electrical Engineering, University of Belgrade, 11120 Belgrade, Serbia; nenad@etf.rs (N.J.); vujo@etf.rs (V.D.)

**Keywords:** autonomous driving, camera, convolutional neural network, deep neural network, embedded systems, end-to-end learning, machine learning

## Abstract

In this paper, one solution for an end-to-end deep neural network for autonomous driving is presented. The main objective of our work was to achieve autonomous driving with a light deep neural network suitable for deployment on embedded automotive platforms. There are several end-to-end deep neural networks used for autonomous driving, where the input to the machine learning algorithm are camera images and the output is the steering angle prediction, but those convolutional neural networks are significantly more complex than the network architecture we are proposing. The network architecture, computational complexity, and performance evaluation during autonomous driving using our network are compared with two other convolutional neural networks that we re-implemented with the aim to have an objective evaluation of the proposed network. The trained model of the proposed network is four times smaller than the PilotNet model and about 250 times smaller than AlexNet model. While complexity and size of the novel network are reduced in comparison to other models, which leads to lower latency and higher frame rate during inference, our network maintained the performance, achieving successful autonomous driving with similar efficiency compared to autonomous driving using two other models. Moreover, the proposed deep neural network downsized the needs for real-time inference hardware in terms of computational power, cost, and size.

## 1. Introduction

Research and development in the field of machine learning and more precisely deep learning lead to many discoveries and practical applications in different domains. The domain where machine learning has a huge impact is the automotive industry and the development of fully autonomous vehicles. Machine learning solutions are used in several autonomous vehicle subsystems, as the perception, sensor fusion, simultaneous localization and mapping, and path planning. In parallel with work on full autonomy of commercial vehicles, the development of various automotive platforms is the current trend. For example, delivery vehicles or various different robots and robot-cars are used in warehouses. The main idea of our work was to develop a solution for autonomous driving for a light automotive platform that has limited hardware resources, processor power, and memory size. Having those hardware restrictions in mind, we are aiming to design a light deep neural network (DNN), an end-to-end neural network that will be able to perform the task of autonomous driving on the representative track, while the developed networks’ model used for inference is possible to deploy on a the low-performance hardware platform.

**Figure 1:**
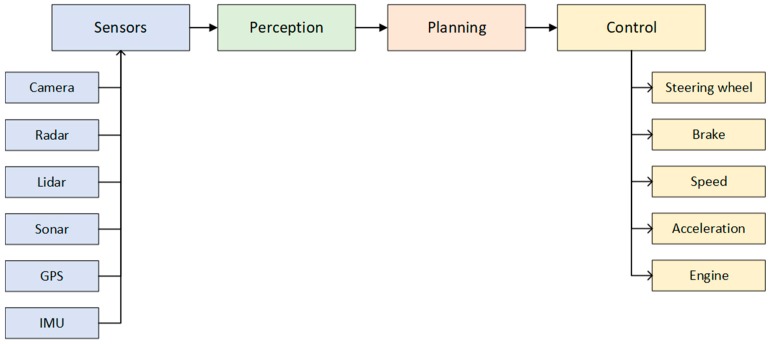
Autonomous vehicle system block diagram.

The autonomous driving system can be generally divided into four blocks: sensors, perception subsystem, planning subsystem, and control of the vehicle, Figure 1. The vehicle is sensing the world using many different sensors mounted on the vehicle. The information from the sensors is processed in a perception block, whose components combine sensor data into meaningful information. The planning subsystem uses the output from the perception block for behavior planning and for both short- and long-range path planning. The control module ensures that the vehicle follows the path provided by the planning subsystem and sends control commands to the vehicle.

**Figure 2:**
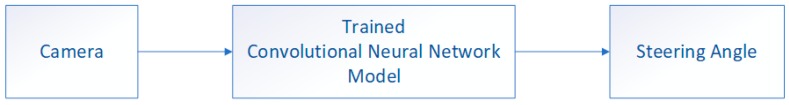
Block diagram of an end-to-end autonomous driving system.

An end-to-end deep neural network we designed for autonomous driving uses camera images as an input, which is a raw signal (i.e., pixel), and steering angle predictions as an output to control the vehicle, Figure 2. End-to-end learning presents the training of neural networks from the beginning to the end without human interaction or involvement in the training process. The purpose of end-to-end learning is that the system automatically learns internal representations of the necessary processing steps, such as detection of useful road characteristics, based only on the input signal.

Nowadays the machine learning applications have been increasingly deployed to embedded devices, mobile phones, and the Internet of Things (IoT) solutions. Deployment of machine learning solutions to embedded hardware platforms leads to new developments in two directions: development of novel hardware platforms able to process data needed for machine learning inference, and development of novel light machine learning architectures and model implementations suitable for low-performing hardware.

The known solutions for end-to-end learning for autonomous driving [1,2,3,4,5] are developed mostly for the real vehicles, where the machine learning model used for inference is deployed on the high-performance computer, which is usually located in the trunk of the vehicle, or those solutions use very deep neural networks that are computationally expensive (e.g., using ResNet50 architecture in) [3]. However, our idea was to develop a significantly smaller solution, a light deep neural network, with similar performance during autonomous driving as known solutions, but using a smaller computational cost that will enable deployment on an embedded platform. This lighter solution will be used for robot-cars, embedded automotive platforms able to carry the goods or perform some similar tasks among relatively known trajectories.

In this paper, we present a novel deep neural network developed for the purpose of end-to-end learning for autonomous driving, called J-Net, which is designed for embedded automotive platforms. In addition to this, for the purpose of objective evaluation of J-Net, we discuss results of our re-implementations of PilotNet [1,2] and AlexNet [6]. AlexNet is originally developed for object classification, but after our modifications, the new AlexNet-like model is suitable for autonomous driving. Firstly, the novel deep neural network architecture J-Net is developed, the model is trained and used for inference during autonomous driving. Secondly, we have compared architectures of three network architectures, J-Net, PitotNet, and AlexNet, and discuss computational complexity. This is done from the reason to have an objective assessment of our network architecture. Next, the implemented models have been trained with the same dataset that we collected. The data collection and inference are done in the self-driving car simulator designed in Unity environment by Udacity, Inc. [7]. Finally, the trained models have been used for the real-time autonomous driving in a simulator environment. The results of autonomous driving using each of these three deep neural network models have been presented and compared. Results of the autonomous driving are given as video recordings of the autonomous driving in a representative track in a simulator environment, in addition to the qualitative and quantitative performance evaluation of autonomous driving parameters during inference.

The results show that while the complexity and size of novel network are smaller in comparison with other models, the J-Net maintained the performance, achieving similar efficiency in autonomous driving. Aiming for implementation on the embedded automotive platform amplifies the importance of a computationally light solution for the DNN used for autonomous driving, since embedded systems may suffer from hardware limitations for onboard computers that are not capable of running state-of-the-art deep learning models.

In the next section, we will present the related work. The autonomous driving system used for data collection and autonomous driving is described in section III. Dataset and data collection strategy are given in section IV. Proposed approach, architecture, and implementation details of J-Net are given in section V. The implementation of models, comparison of network architectures and parameters, and the training strategy for all three solutions are presented in section VI. Results and discussion of the implementation of all three neural networks and inference during autonomous driving are given in section VII. The conclusion is given in the last section.

## 2. Related Work

Deep learning is a machine learning paradigm, a part of a broader family of machine learning methods based on learning data representations [8,9,10,11]. Representations in one layer of a deep neural network are expressed in terms of other, simpler representations from previous layers of the deep neural network. The core gradient of deep neural networks are convolutional networks [12]. Convolutional neural networks (CNN) are a specialized kind of neural network for processing data that has a known grid-like topology. CNNs combine three architectural ideas: local representative fields, shared weights, and spatial or temporal sub-sampling, which leads to some degree of shift, scale, and distortion invariance. Convolutional neural networks are designed to process data with multiple arrays (e.g., color image, language, audio spectrogram, and video), and benefit from the properties of such signals: local connections, shared weights, pooling, and the use of many layers. For that reason, CNNs are most commonly applied to analyzing visual imagery.

**Figure 3:**
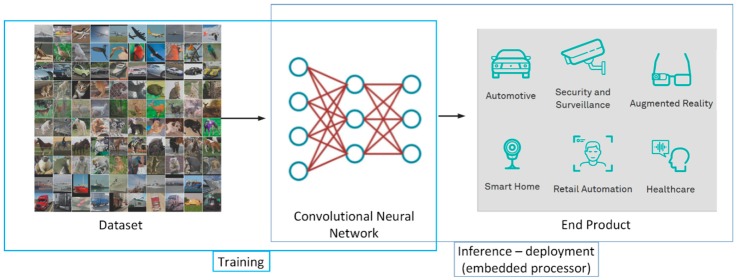
General deep learning development flow. Training—once the convolutional neural network model is developed it is trained with the appropriate dataset. Inference—the trained model is deployed at the end product and used for inference with real-time input data.

Deep learning for computer vision has significant usage in various commercial and industrial systems and products, as automotive, security and surveillance, augmented reality, smart home applications, retail automation, healthcare, and the game industry; Figure 3. Convolutional neural networks were some of the first deep models to perform well and were some of the first networks to solve important commercial applications. One of the first examples of convolutional neural network practical application was in the 1990s by research group at AT&T, Inc. that uses CNN for optical character recognition and reading checks [13]. Later, several optical character recognition and handwriting recognition solutions were developed based on convolutional neural networks [14], while the newest applications of CNNs for computer vision are endless [15,16,17,18].

Significant contribution to the development of convolution neural networks and deep learning architectures is given by ImageNet Large Scale Visual Recognition Challenge [19]. Over several years, the architectures that won this competition represent the state-of-the-art of neural networks and deep learning, becoming a building block and inspiration for new solutions. Some of the most known architectures are AlexNet designed by the SuperVision group from University of Toronto [6]; VGG-16 model designed by VGG (Visual Geometry Group) from University of Oxford [20]; GoogLeNet designed by researches from Google, Inc. [21] that introduced inception modules; Residual Neural Network (ResNet) designed by researchers from Microsoft Research [22], with the depth of even 152 layers; and ReNet designed by researches from Politecnico di Milano and University of Montreal [23]. Some of the novel breakthroughs in deep learning are automated machine learning [24], training deep networks with synthetic data [25], video-to-video synthesis [26], playing the game of Go [27,28], and end-to-end learning [29,30,31].

The first successful attempts of the development of autonomous vehicles started in the 1950s. The first fully autonomous vehicles were developed in 1984 [32,33], and in 1987 [34]. Significant breakthrough in the field of autonomous vehicles is done during the Defense Advanced Research Projects Agency’s (DARPA) challenge, Grand Challenge events in 2004 and 2005 [35,36], and Urban Challenge in 2007 [37], where it was demonstrated that machines could independently perform the complex human task of driving. 

Although there are the prototypes of autonomous vehicles currently tested on the regular streets, some of the challenges for the autonomous driving are not completely solved yet. Current challenges in autonomous vehicles development are sensor fusion [38,39,40,41], higher-level planning decisions [42,43,44,45,46], an end-to-end learning for autonomous driving [1,2,3,4,5,47,48,49], reinforcement learning for autonomous driving [5,50,51,52,53], and human machine interaction [54,55]. A systematic comparison of deep learning architectures used for autonomous vehicles is given in [56], a short overview of sensors and sensor fusion in autonomous vehicles is presented in [57]. 

The aim of our work was to achieve an end-to-end learning using only camera images as an input. Although there are many sensors used for autonomous vehicles, such as lidar, radar, sonar, a global positioning system, an inertial measurement unit, and wheel odometry, the camera is an indispensable sensor in autonomous driving, which enables an autonomous vehicle to visualize its surroundings. Cameras are very efficient at the classification of texture interpretation, widely available, and more affordable than other sensors used for detection, such as radar or lidar. The limitation of the camera is the computational power needed for processing the data. 

In this paper, we present a novel network for simplified solution of an end-to-end learning for autonomous driving. The input in our autonomous driving system is only the camera image, the raw pixel. Output is steering angle prediction. The aim is to achieve the computationally light model that can be deployed to an embedded automotive platform for real-time inference. Developing a non-expensive machine learning solution in terms of computational power and memory resources is not easy to achieve [58,59,60,61,62]. The techniques that enable efficient processing of deep neural networks to improve energy efficiency and throughput without sacrificing application accuracy or increasing hardware cost are critical to the wide deployment of deep neural networks in artificial intelligence systems [62].

The goal of machine learning algorithms is to solve the problem they are addressing with the highest possible accuracy. It often leads to very complex and deep neural networks that are computationally demanding [60,61]. This is especially the case for deep learning for computer vision-based applications. For example, some of the well-known models that use a large number of layers in network architecture are VGGNet (16 to 19 layers) [20], GoogLeNet (22-layerd inception architecture) [21], ResNet (152 layers) [22], and similar network architectures based on those. Finally, the progress in convolutional neural networks development and applications, and experimentation with more complex architectures are a consequence of two factors, having a large amount of data and improved computational efficiency.

However, application on real-time embedded platforms with limited computational power and memory spaces seeks for a different approach [62]. Since the execution of DNN depends heavily on the weights, also called model parameters, the solution for deep neural network architecture suitable for applications on embedded platforms is the smaller model that needs to communicate less data [58]. This is challenging to achieve, especially if the application is computer vision and that has, as an input, a high-quality image. The reduction of the neural network depth and number of parameters often leads to accuracy degradation. Hence, by developing deep neural networks for computer vision for embedded platforms we are looking for a solution that will be good enough for both an acceptable accuracy and the possibility for inference on hardware platforms with limited capabilities. Therefore, our goal is to develop the right deep neural network architecture that can achieve acceptable accuracy, the successful autonomous driving in a representative track, but which operates in real-time within power and energy limitations of its target embedded automotive platform.

In order to achieve this, the approach we took was not to reduce the parameters of some of the well-known neural networks that can be used for autonomous driving, but to start from scratch, developing a network architecture layer by layer, until we found the satisfying solution. The general workflow to find an appropriate model size is to start with relatively few layers and parameters, and to increase the size of the layers or add new layers until returns diminish with regard to validation loss.

In summary, the contributions of the approach proposed in this paper are:

1. We modified the convolutional neural network for image classification, AlexNet, and used the new AlexNet-like model for end-to-end learning for autonomous driving.
2. We proposed a novel deep neural network J-Net that, despite having a light architecture in comparison with AlexNet and PilotNet, is able to successfully perform autonomous driving. This recommends the J-Net model for deployment on low-performing embedded automotive platforms.
3. We have demonstrated the suitability of a new proposed network J-Net for autonomous driving for embedded automotive platforms by doing the performance evaluation of autonomous driving in simulated environment, where the J-Net shows the best performance in terms of latency and frame rate, among three implemented solutions.

## 3. Autonomous Driving System

**Figure 4:**
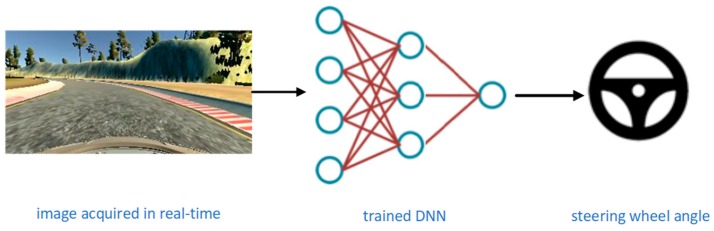
Real-time autonomous driving—the image acquired from the central camera is fed to the trained deep neural network (DNN) model and the output of this model is the steering angle prediction that controls the vehicle.

In our approach, we used end-to-end learning for an autonomous driving system. Input in our autonomous driving system was the image, raw pixels, and the output was the control of the vehicle, the steering angle. The end-to-end learning was applied whereby the network will learn how to control a vehicle only based on an input signal from the camera during real-time inference, Figure 4.

**Figure 5:**
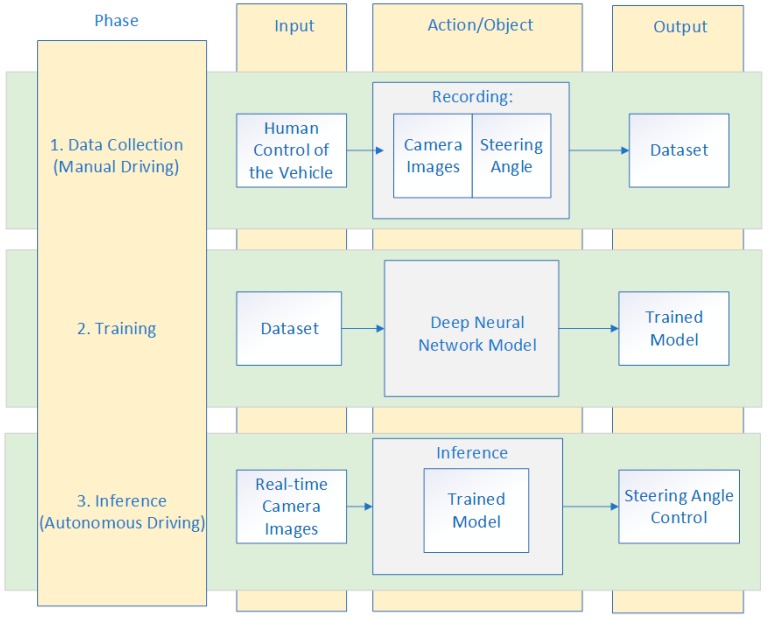
Block diagram of the autonomous driving framework.

Firstly, in order to collect the data that would be used for training the end-to-end DNN model, a human driver was driving the vehicle and simultaneously recording the images and steering measurements. If the simulator environment for autonomous driving was used, the vehicle was driven in manual (training) mode by a human driver using a keyboard, mouse, or joystick, and the dataset was automatically collected. Data acquired during manual driving mode were camera images and the steering angle values per each frame. The images were used as the feature set, and the steering measurements as the label set. The speed of the vehicle was fixed due to simplicity. Data collected using this approach were used for training the neural network that will learn to drive based only on the input data, without any further human interaction. This technique is also known as behavior cloning. Secondly, the deep neural network for autonomous driving was trained on this dataset to predict the steering angle. Finally, the trained model was used for inference, a real-time autonomous driving in the same simulator environment. The metrics of successful driving on the representative track was that the vehicle remains on the track at all times during autonomous driving. A block diagram of the autonomous driving framework that we used is presented in Figure 5.

### Simulator Environment

**Figure 6:**
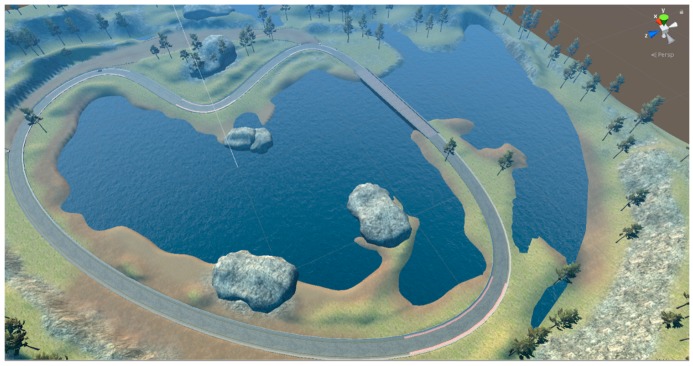
Top view of the scene in Unity editor for the self-driving car simulator [7].

The platform used for data collection and inference, evaluation of successful autonomous driving was a self-driving car simulator [7]. The self-driving car simulator is built in the Unity game development environment. Top view of the representative track used for autonomous driving is presented in Figure 6. 

**Figure 7:**
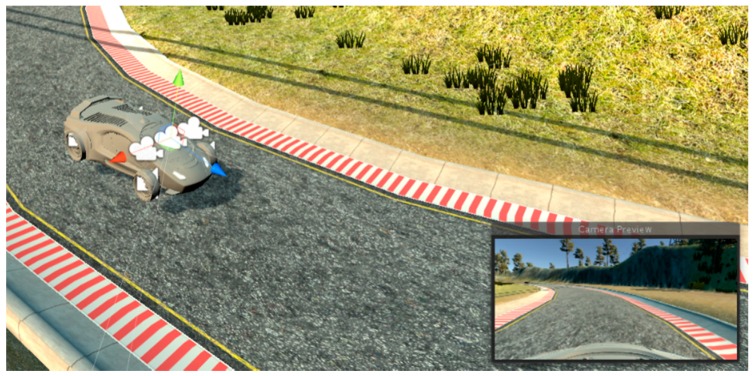
The vehicle in the simulator on the representative track, with three cameras on top of the vehicle. In right bottom corner is the view from the central camera at this particular moment.

The representative track for autonomous driving was used for data collection. While the car was driving in manual mode, the images from the cameras mounted on top of the car were recorded together with the steering angle for that frame, Figure 7. The data from the all three cameras mounted on top of the vehicle were recorded and stored together with the information about steering angle for the same frame. The same representative track was used for driving in the autonomous driving mode where the decision about steering angle was made by a real-time image from the camera mounted on the vehicle.

**Figure 8:**
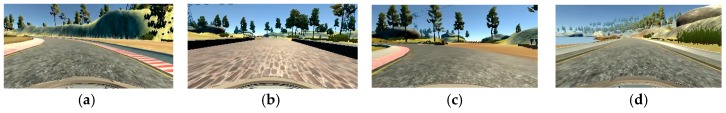
Input data captured in different frames using the central camera. During the representative track, the different road characteristics and the borders are presented. The four main track borders and road characteristics are: (**a**) red and white stripe—mostly sharp curves; (**b**) a small wall—the bridge with different road texture; (**c**) red and white stripe on one side and dirt on the other side—curves; (**d**) shoulders—mostly straight road.

Different features of the representative track present challenges for training an end-to-end autonomously driving model. For example, the model has to learn how to handle sharp turns, different textures, and different borders of the road. The most challenging parts of the representative track for autonomous driving were three right angle curves right after the bridge, as can be seen in Figure 6. The track is defined with a red and white stripe, a shoulder, or just the dirt as a border between track and rest of the simulator environment. Examples of images recorded with the central camera in different frames, which present different road characteristics, are presented in Figure 8. The part of the track defended with a red and white stripe is presented in Figure 8a. Different texture of the road can be seen in Figure 8b, that presents the bridge on the lake. On the bridge, the road is tiled with bricks, while on the other track parts it is mostly the paved road. Moreover, the border of this part of the track is a low wall. The parts of the track defined with dirt (at one side) and the parts defined with shoulders are presented in Figure 8c and Figure 8d, respectively. These different features of the road in a simulator environment aids better generalization of the model that led to successful autonomous driving in different scenarios. 

## 4. Dataset

### 4.1. Data Collection

**Figure 9:**
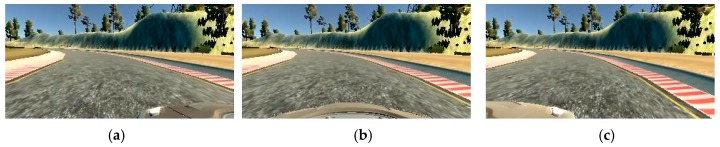
Example of data collection taken simultaneously using the three cameras mounted on the vehicle: (**a**) left; (**b**) center; (**c**) right camera. This is an example of images captured in the very first frame.

Data collection was done while the vehicle was driving in manual mode on the representative track. Image data were acquired from the three cameras mounted on the vehicle in the simulator environment, as shown in Figure 7. At the end of the manual ride, the images were stored together with the table containing information about image titles and steering angle values per each recorded frame. An example of images recorded by all the three cameras in one frame is presented in Figure 9. Three cameras were used for training purpose. During the data collection, in each frame, the images from three cameras were captured with the same steering measurement value. The slight difference in the field of view per each central, left, and right camera leads to a better generalization of the model. 

In order to have good quality samples, that will enable the model to learn the right features, the vehicle should be driven in the manual mode the same way we expect it to autonomously drive during inference. During the data collection, the aim was that the vehicle was driven in the center of the road. Special attention was given during driving in the right-angle curves. It was very important that our model learnt how to behave in the curves. 

### 4.2. Data Augmentation

For the data acquisition, several laps were recorded while driving in the manual mode, where the data from three cameras were collected. Even after several laps, the collected dataset was relatively small. Therefore, we applied data augmentation techniques. One of the most important features that our neural network had to learn was the curves. A simple method to double the dataset size and put the focus on curves was data augmentation, where the images were flipped in vertical access and the steering angle was multiplied with −1. 

In addition to doubling the number of training data, the data augmentation by flipping images provided additional value to the final model. Since the predefined track is a closed loop, there are more curves in one direction than in the other. In our case, there were more curves on the left. If we would use only the collected data without data augmentation using flipping images, the model would learn to steer on the left even if the ground truth for that frame would be to stay straight. Data augmentation by mirroring images using a horizontal flip, and inverting the steering angles, provided balanced datasets where the model was thought to steer both clockwise as well as counter-clockwise.

### 4.3. Dataset

The total number of acquired samples was 34,288 images with a resolution of 320 × 160 × 3. Each recorded image was 320 pixels high, 160 pixels wide, and three channels deep—a color RGB image. The average memory size of one recorded image was about 13.5 kB. Each image was paired with the corresponding steering angle value that was normalized in range between −1 and 1. After applying data augmentation, the total number of samples in our dataset was 68,576. The data was split into training and validation categories, where 80% of data was chosen for training, 54,861 samples, and 20% of the data for the validation, 13,715 samples; Table 1. 

Testing was done in real-time during inference. Real-time acquired images from the central camera were continuously sent to the trained machine learning model that was used for the control of the vehicle steering angle. Evaluation of the successful model was done by observating if the vehicle was able to drive autonomously during the entire predefined track. If the vehicle was off of the road, it was considered as unsuccessful autonomous driving. 

### 4.4. Data Preprocessing

For training the deep neural network, images acquired from all three cameras were used—central, left, and right; Figure 9. Using three cameras for gathering data for training deep neural network for autonomous drive was inspired by [1]. All of these images captured a similar scene but from slightly different positions. Advantages of using three cameras instead of one central camera are three times more data, and better performance for steering back to the center scenario when the vehicle starts drifting off to the side. The steering angle was paired with three images from a particular frame corresponding to the central camera, and the images from the left and right cameras had a field of view shifted on the left or right side of the road, respectively, which means that the steering angle for the left and right images is not correct. To overcome this, the correction factor for the steering angle measurement had been added or subtracted from the left and right images, respectively. The correction factor was one of the hyperparameters to be fine-tuned during the training process.

**Figure 10:**
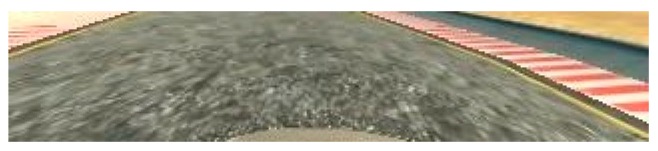
Input image after the cropping.

Cropping was used in order to remove the parts of the image that do not have valuable information for autonomous driving, so as to remove the sky threes and hills on the top of image and the part of hood of the vehicle on the image bottom. An example of the image after cropping is presented on Figure 10.

The images from dataset were normalized by dividing each pixel of the image by 255, which is the maximum value of an image pixel. Once the image was normalized to a range between 0 and 1, mean centering of the data was applied by subtracting 0.5 per each pixel:

(1) $$x_{norm}=\frac{x}{255}-0.5$$

that resulted with −0.5 < $x_{norm}$< 0.5.

## 5. Proposed Approach

The leading idea during the design process was to achieve end-to-end autonomous driving using the light model (computationally least expensive model), while simultaneously achieving the best possible performance, in our case the autonomous driving on the representative path. The computationally least expensive model is usually the model with the least number of parameters that effects their memory footprint and computations. Therefore, the type and size of layers, kernel sizes, and a number of feature maps have an influence on computational performance. The performance of autonomous driving was examined in a self-driving car simulator.

**Figure 11:**
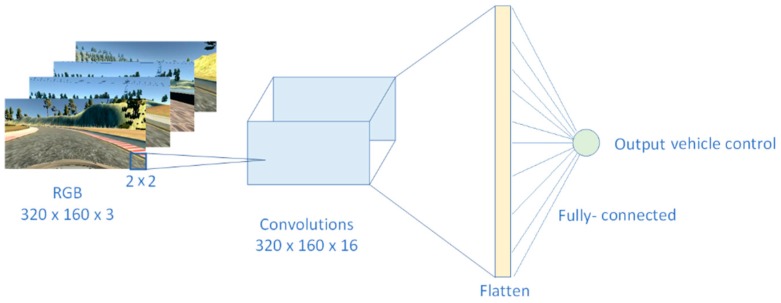
Architecture of experimental shallow deep neural network (DNN) with just one convolutional layer with 32 feature maps, one flattened layer and, at the end, the fully connected layer.

The final architecture of the J-Net model was the result of experimenting with building blocks of deep neural networks—different number of layers, kernel sizes for convolutional layers, number of feature maps, placement of pooling layers, and, at the end, experimenting with the size and number of the fully-connected layers. Block diagram of this experimental shallow CNN is presented in Figure 11.

The first step in design of the novel solution was to use an extremely shallow CNN; we performed a 2D convolution operation on the raw data of the input image:

(2) $$S\left(i,j\right)=\left(I*K\right)\left(i,j\right)=\underset{m}{\sum }\underset{n}{\sum }I\left(i-m,j-n\right)K\left(m,n\right)$$

where the three-channel input image *I* had the dimensions 320 × 160, the used two-dimensional kernel size was 2 × 2. The kernel was used to extract the patches of the image in convolutional operation. The output of the convolution operation was *S*(*i*,*j*), the two-dimensional feature map tensor. The weights, *w*, were shared across patches for a given layer in a CNN to detect the particular representation regardless of where in the image it was located. The equation to calculate width and height of the convolutional output layer is:

(3) $$\begin{array}{c} W_{out}=\frac{W-F+2P}{S}+1\\ H_{out}=\frac{H-F+2P}{S}+1 \end{array}$$

where *W* and *H* are width and height of input layer, *F* is filter (kernel) size, *S* is stride, *P* is padding, and *K* is number of filters. In our first experiment, input image was 320 × 160, *F* = 2, stride is 1, and we did not use padding, *P* = 0. This led to the size of output layers: $W_{out}$ = 319, and $H_{out}$ = 159. Output depth was equal to the number of filters; $D_{out}$ = 16 in this case. Output volume after convolutional operation is:

(4) $$W_{out}*H_{out}*D_{out}$$

which was 811,536. After convolution, the *ReLU* activation function was applied:

(5) ReLU(x)=max(0,x).

*ReLU* activation function is the most commonly used activation in hidden layers of DNN. The main advantage of *ReLU* over other activation functions, such as *sigmoid* or *tanh*, is that with *ReLU* there is no problem with gradient vanishing [59].

This convolution was followed with the flattened layer, where the two-dimensional feature map was converted into a single dimension vector. The flattened layer did not result with the new trainable parameters, since the conversion of nodes from the previous layer into a single dimension. Finally, at the end, when we had spatial features of an image as a result of convolution, we applied fully-connected layer where the all nodes from the flattened layer were combined into the single output, which directly predicted a steering angle values. Since there is single output node from the network, this is a regression network.

The model was trained with the dataset previously collected, using mean squared error (MSE) loose function and Adam optimizer, as described in Section 6.2. The results during real-time inference, as expected, was not good, the car was not able to maintain course on the road. However, qualitative performance evaluation showed that the model still learned some useful features. As it can be seen from the video of autonomous driving in the simulator environment [63], the model learned to follow the line, so the vehicle was driving by the edge of the lake, following the line between the ground and the water. This little experiment showed that the chosen direction was appropriate, but the model needed more features to be extracted in order to have successful autonomous driving.

As it can be seen from the first experimental results, even though the network we used was very shallow, with only three layers, the number of nodes and weights and the trainable parameters was quite high. The reason for this is that we used the entire input image for convolution, which produced a high number of nodes in the output layer, and we did not use any operation for reduction of parameter numbers, like the pooling operation. Insights from this experiment were valuable for the next phase of development of the final solution for our light deep neural network for autonomous driving:

1. Do not use the entire input image size. Parts of the image as the sky or the lower part of the image was not relevant for the autonomous driving. What is relevant is the road, the curves, and the boundaries of the road, like a red stripe, shoulder, dust, bridge.
2. Do perform data normalization in order to have the same range of values for each of the inputs to the model. This can guarantee stable convergence of weight and biases.
3. Use the pooling operation in order to lower the number of network nodes in the next layer, and, consequently, the number of trainable parameters. A pooling layer is generally used to decrease the size of the output and to prevent overfitting.
4. Use more convolutional layers, since convolutions are responsible for feature extractions. The first experiment showed that the convolutional layer can extract some features needed for autonomous driving (e.g., one feature is a line to be followed by the autonomous vehicle), but one convolutional layer was not good enough for our aim, more feature maps are needed.

### The J-Net Architecture

Introducing more hidden layers to a deep neural network helps with parameters efficiency. It is likely to get much more performance with pure parameters by going deeper rather than wider. In addition to this, deep neural networks applied to images are very efficient, since images tend to have a hierarchical structure that deep models naturally capture. The lower layers of deep neural networks capture simple features like lines or edges. Further layers extract more complicated features like geometric shapes, and the last layers are extracting objects. Since the aim of our work was to drive a vehicle in a representative track, the features needed to be extracted were not objects, rather they were the simple features or geometric shapes. For that reason, for our final model, we have chosen three convolutional layers followed with one flattened layer and two fully connected layers, as will be discussed in more detail in the further text. 

**Figure 12:**
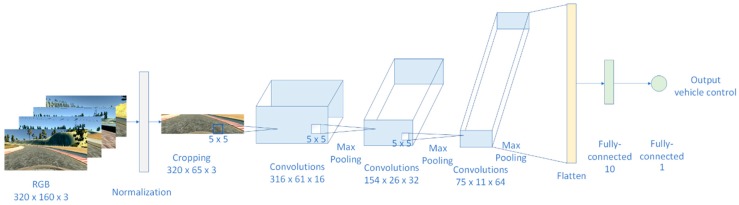
Architecture of proposed end-to-end deep neural network, J-Net, used for autonomous driving. The network has three convolutional layers with 16, 32, and 64 feature maps, one flattened layer, and two fully-connected (dense) layers. Max pooling is placed after every convolutional layer.

For the reason of better features extraction, we have chosen three convolutional layers in order to extend extracted features; Equation (2). We chose three convolutional layers with 16, 32, and 64 feature maps, respectfully; Figure 12. On the input to the first convolution the size was 320 × 65 × 3, after normalization and cropping of the raw image, then we applied the kernel size of 5 × 5 with 16 feature maps. Based on Equations (3) and (4), the total number of trainable parameters after the first convolutional layer was 1216. 

Since we wanted to achieve a light solution, and convolution is a very expensive operation that adds a significant number of the network nodes, and the weights assigned to each of those nodes, downsampling was needed. One solution for this problem would be using striding during convolution, to shift the filters by a few pixels each time and reduce the feature map size. However, this downsampling of an image may cause the loss of some important features since it removes a lot of information. The second solution on downsampling an image is the pooling operation. Instead of skipping one in every two convolutions, we used a small stride in combination with all the convolutions in the neighborhood and combined them. In order to reduce size of the deep neural network layers, we applied the max pooling operation after each convolutional layer. In max pooling layer, every point of the feature map is compared with a small neighborhood around that point and the maximum of all the responses around it is computed:

(6) $$y=max\left(X_{i}\right)$$

where $X_{i}$ is the value of one input point.

The first advantage of using max pooling is that this operation is parameter free, it does not add new parameters. This lowers the possibility of having increased overfitting. Second, max pooling often yields a more accurate model. On the other side, since the convolutions that went below run at the lower stride, the model becomes more expensive to compute. Additionally, introducing a new layer as pooling adds more hyperparameters to tune, such as the pooling region size and the pooling stride. The pooling layer operates independently on every depth slice of the input and resizes it spatially. In our model we chose MaxPooling with size 2 × 2 that downsamples every depth slice in the input by 2, along both width and height, discarding 75% of the activations. The depth dimension remained unchanged. In this case, we reduced the number of trainable network parameters, while the feature map was not degraded much. 

After applying the MaxPooling layer after the first convolution, the input size of the second convolutional layer was 158 × 30 × 16, and the second kernel size was 5 × 5 with 32 feature maps, which, after calculating number of trainable parameters using Equations (3) and (4), led to 12,832 trainable parameters for the second convolution. After this convolution layer, the same MaxPooling as after the first convolution was applied. The third kernel size was 5 × 5 with 64 features map, which led to the total number of trainable parameters of 18,496, after the third convolution.

The final solution for J-Net had three convolutional layers, each followed by the ReLU activation function described in the previous subsection, followed with the MaxPooling layer size of 2 × 2. Applying convolution and MaxPooling operations led to 32,544 trainable parameters after the last convolutions. Since we were developing the representative learning network, those nodes had to be connected to the one final node. After the last convolutional layer, the flattenedlayer was added to get a one-dimensional vector of parameters. The flattened layer did not add new parameters, but, rather, only rearranged the existing in one dimension. Finally, the last layers of the developed DNN were two fully connected layers, the first with ten output nodes and the second with just one output node for the steering angle prediction. The final number of trainable parameters for this architecture and for the input size of 320 × 160 × 3 pixels was 150,965. The network architecture is presented in Figure 12.

## 6. Implementation

In order to make an objective performance evaluation of our network, we re-implemented three neural network models: LeNet-5 [13], AlexNet [6], and PilotNet [1], with small modifications in order to be able to aid end-to-end learning for autonomous driving. The idea was to have an objective evaluation of our model, J-Net, in comparison with known network architectures. For that reason, all implemented models were trained with the very same dataset we created, which is described in Section 4, and tested in the simulator using the same conditions. Unfortunately, autonomous driving using the LeNet-5 model was not successful, the vehicle was not able to remain on the track, so this model was excluded from further examination. Inference using AlexNet and PilotNet was successful in autonomous driving during the whole track. 

**Figure 13:**
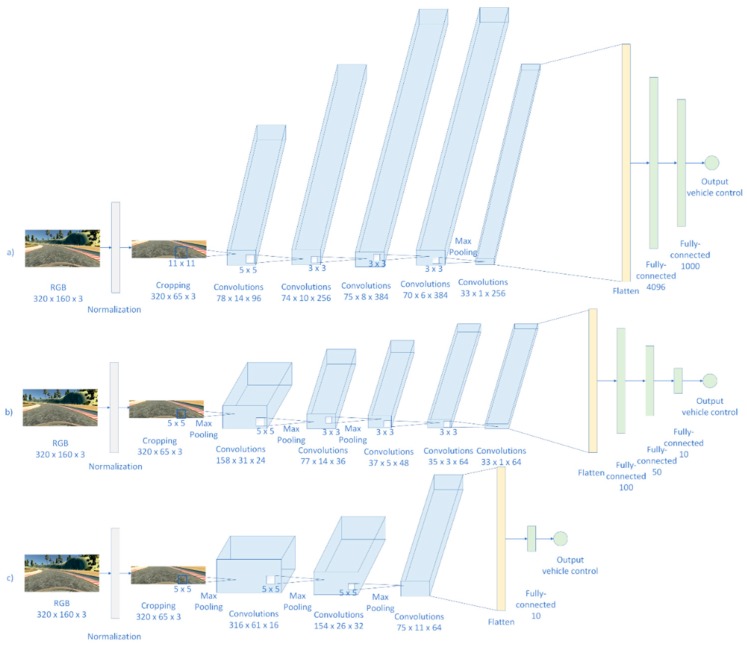
Comparison of deep neural network architectures that we implemented and used for end-to-end autonomous driving: (**a**) AlexNet; (**b**) PilotNet; (**c**) J-Net.

As the J-Net architecture is described in previous chapter, here we will discuss implementation details of AlexNet and PilotNet re-implementations. The network architecture comparison of J-Net and AlexNet and PilotNet is shown in Figure 13.

### 6.1. Design Details for AlexNet and PilotNet Re-Implementations

In our work, the AlexNet architecture [6] was re-implemented and adapted for the purpose of end-to-end learning for autonomous driving. In the original architecture of the AlexNet, there are two parallel pipelines of processing. This is from the historical reasons, the original AlexNet was trained using two graphics processing units (GPUs), and that is the reason why they split convolutions in two parallel branches. Since we had enough hardware resources to train AlexNet using one GPU, in our implementation of AlexNet we simplified the architecture and used one stream of convolutions, using a combined number of filters proposed by the original solution. The other differences between the original architecture and our implementation of that architecture was the size of the input information, for AlexNet it was 224 × 224 pixels, for our case the input image was 320 × 160 pixels, and after cropping, which was needed to dismiss unneeded information contained in edge pixels, the image size that our implementations were dealing with was 320 × 65 pixels. Difference in input layers influenced that in our reimplementation of AlexNet, so that we omitted two pooling layers. The introduction of all three pooling layers would significantly reduce the number of pixels available for the next convolutions, and since we had a smaller input image in one dimension, there would not be enough pixels for all convolutional layers with usage of original kernels. In order to simplify and modify the network for our purposes, we omitted the first two pooling layers from the original network, and had one max pooling layer after the forth convolutional layer, with 384 features, and before the last convolutional layer, with 256 features. 

Difference in input images, between original architecture and our re-implementation, influenced the total number of parameters. In our case we had a lower number of trainable parameters than was in the original architecture. The original AlexNet has over 63 million trainable parameters, while our re-implementation of AlexNet had over 42 million trainable parameters. The last difference between original architecture and our implementation of AlexNet is the last layer. In the original AlexNet there were 1000 nodes as the output layer, since on ImageNet competition there was 1000 classes. In our application, just one output node was needed: steering angle prediction for the vehicle control.

PilotNet, also called NVIDIA CNN that is designed by researchers from NVIDIA Corporation, is the deep neural network for end-to-end autonomous driving, created with the motivation to improve the DARPA Autonomous Vehicle (DAVE) system for autonomous driving [1,2]. PilotNet has a normalization layer, followed with five convolutional layers, followed by four fully connected layers. Difference between original PilotNet architecture and our PilotNet implementation is the input of the network, which led to the slight difference is size of the layers and number of trainable parameters. In the original PilotNet the input plane is 200 × 66 pixels, while in our case the input was 320 × 160 pixels, and after cropping it was 320 × 65 pixels. Order of the remaining convolutional, flattened, and fully-connected layers was similar, with the same numbers of features maps for the convolutional layers. Since the size of the original input to the PilotNet was not the same as our input size, there was a difference in the number of trainable parameters. For example, the flattened layer, after the fifth convolutional layer, in the original PilotNet architecture has 1164 neurons, while in our implementation of PilotNet there were 2112 neurons. Except this, our re-implementation of PilotNet was following the original model.

### 6.2. Training Strategy and Hyper-Parameter Tuning

The three architectures J-Net, AlexNet, and PilotNet were implemented in Python using Keras [64], a high-level deep neural network library, which is written on top of the TensorFlow library. We implemented and trained all networks from scratch. The platform used for training and interfacing was the PC with a 12-core processor on 3.2 GHz and the NVIDIA graphics processing unit GeForce GTX 1070-Ti with 8GB GDDR5 and 8Gbps memory type-speed.

**Figure 14:**
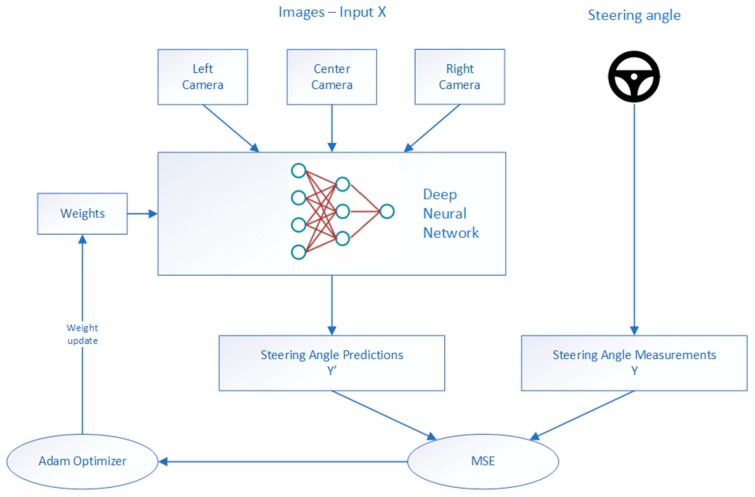
Training flowchart. Data collection is done in manual driving mode by acquiring images from three cameras mounted on the vehicle: central, left, and right in parallel with recording the steering angle paired with each image. During the training process, the correction factor has been added to the left and right images.

During training, the left and right camera images have been feed to the model as if they were coming from the center camera; Figure 14. Using three cameras tripled the data collected and helped the model to learn how to steer if the car drifts off to the left or the right. Images from the central camera were taken without charges, while for the images from the left and right cameras, the correction parameter was applied to the steering angle measurements, as described in Section 4.4. After fine tuning, we chose the correction factor 0.22 to be added or subtracted from the left and right images, respectively. In addition to this, data preprocessing that was applied here included cropping images, data normalization, and mean centering the data, as explained in Section 4.4. For normalization, we used the Lambda layer from the Keras library. 

Each of the designed models were trained separately using the dataset previously collected. For the loss function, the mean squared error was used to minimize the error between the steering prediction and the ground through steering measurements. MSE was chosen since it is an appropriate loss function for the regression networks [8,9,10]. MSE takes the average of the square of the difference between the original values and the predicted values. The advantage of MSE is an easier gradient computation. Computation of square of the error leads to the effect of larger errors becoming more pronounced then smaller errors, hence the model is focused more on the larger errors:

(7) $$MeanSquaredError=\frac{1}{N}\overset{N}{\underset{j=1}{\sum }}{\left(y_{j}-{\hat{y}}_{j}\right)}^{2}$$

where the ${\hat{y}}_{j}$ is the vector denoting values of *N* number of predictions. Additionally, $y_{j}$ is a vector representing *N* number of true values.

During the network training, the Adam optimizer [65] was applied for all three models. Adam optimization is one of the most effective optimization algorithms for training deep neural networks. Empirical results demonstrate that Adam works well in practice and compares favorably to other stochastic optimization methods. Loss values during the different epochs during training of all three models is shown in Figure 14.

**Figure 15:**
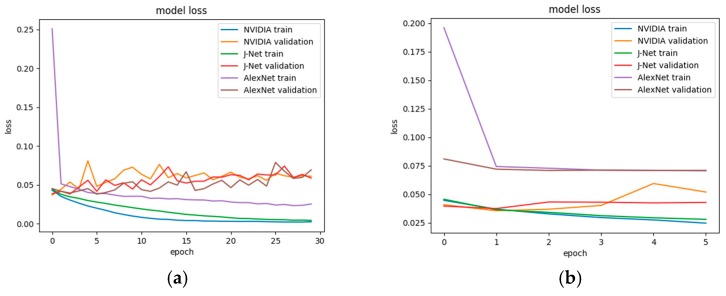
Model loss for training and validation for AlexNet, PilotNet (i.e., NVIDIA CNN—Convolutional Neural Network by NVIDIA Corporation), and J-Net (**a**) for training in 30 epochs; (**b**) for training in 6 epochs.

Since we were using a relatively small dataset, there was a possibility that overfitting will occur. Therefore, the early stopping regularization method was applied. Developed models of J-Net, AlexNet, and PilotNet deep neural networks were trained with a different number of epochs. In the beginning, the models were trained with 30 epochs, Figure 15a. However, only for the AlexNet, this number of epochs gave a good result; the loss for validation was decreasing during the same time as a loss for the training. AlexNet showed good performance in autonomous driving, by completely finishing the task of end-to-end autonomous diving in the simulator. On the other hand, training of PilotNet and J-Net with 30 epochs showed overfitting, the validation loss started to increase while the training loss continued to go lower. Using this number of epochs for training PilotNet and J-Net, the vehicle was autonomously driving successfully at one part of the road, but after the car got into the curve, the vehicle went off the road. In order to prevent overfitting, firstly, we tried the dropout method, applied after the first dense layer in network architectures. However, this only partly solved the problem since the vehicle was not able to drive the full path. 

In the final solution, an early stopping regularization method was applied. As it was expected, the early stopping provided smaller validation loss values for J-Net and PilotNet models, while the validation loss value for the AlexNet remained on a similar level. Since the used dataset for training was the same for all models, the difference in ratio between testing and validation loss per model trained with a different number of epochs was directly related to the network architecture itself. Both J-Net and PilotNet models had a smaller number of trainable parameters than AlexNet. Therefore, for the same dataset size, it was more likely that overfitting will occur for those two models. Once the early stopping regularization method for overfitting was applied, the J-Net and PilotNet models had an expected reaction, reduction of overfitting, and smaller validation loss; Figure 15b. 

In case of PilotNet, in both cases—30 epochs and 6 epochs—it can be seen from Figure 15a,b that there was a peak of validation loss in the fifth epoch, a notable difference between validation and training loss that indicated the need to apply an early stopping method and to choose four epochs for training the PilotNet. Validation of the model during the autonomous driving confirmed this conclusion, showing that the PilotNet provided the best driving performance when it was trained with four epochs. On the other side, the choice of six epochs for the J-Net model training was the more empirical choice. For training the J-Net model, the experiments showed that the validation loss was gained with a smaller number of epochs, but that number could vary between four and 10 epochs with similar results. We chose six epochs, which provided successful autonomous driving during the validation of the model. All applied hyperparameter tuning techniques led to finalizing the implementation and training of the networks and to the successful completion of the task of autonomous driving in the representative track in a simulator environment.

The trained model was saved and used later for inference for autonomous driving. As it was expected, based on the total number of trainable parameters described in the previous section, the lightest model was J-Net with only 1.8 MB. The model that required the most memory space was AlexNet with 509.5 MB, with is in correspondence with its number of trainable parameters of the untrained network, over 44 million parameters. The trained PilotNet model had a 4.2 MB memory size.

## 7. Results and Discussion

The proposed deep neural network J-Net was compared with AlexNet and PilotNet, which we re-implemented in order to conduct an objective performance evaluation of the novel design. The models of all three network architectures were implemented, trained with the same dataset, and trained models were used for inference in the simulator for autonomous driving. The results were compared in terms of performance, successfully driving on the representative track, and in terms of complexity of the network, a number of trainable parameters, and the size of the trained model. 

### 7.1. Computational Complexity

The execution of the deep neural network models depends heavily on a set of static constants, weights, also called model parameters. The network architecture itself, the connections between nodes, directly determines the computational cost of the network. One of the main differences between conventional neural networks and conventional artificial neural networks is that the connections between the neurons are not fully connected. Hereby, the particular organization of the deep neural network and the precise characterization of the computations in filter elements determines the network complexity. 

In order to have a quantified measure of the computational demand of each trained network, the complexity of the network, the numbers of trainable parameters were compared. The depth of the network, number of layers, and the types of the layers, convolutions, pooling, and density uniquely determine the number of network parameters. This applies to a design model that is not yet trained. In Table 2, the layers and the number of the trainable parameters of the implemented neural networks are presented. As it can be seen from the table, AlexNet had 42,452,305 trainable parameters, PilotNet had 328,219 of trainable parameters, while J-Net had only 150,965 trainable parameters. The network introduced in this paper, J-Net, had about half the trainable parameters than PilotNet, and about 280 times less than AlexNet. In addition to this, during the training, we calculated a number of floating-point operations for each model that were, as expected, proportional to the number of parameters: 42.45 million multiplication and the same number of addition operations for the AlexNet, 347.82 thousand operations for multiplication and for the addition for the PilotNet, and about 150.84 thousand operations for the multiplication and for the addition for the J-Net; Table 2.

Additionally, we compared the size of the trained models: The AlexNet model had a memory size of 509.5 MB, PilotNet 4.2 MB, and J-Net only 1.8 MB; Table 3. All models were trained with the same dataset, loss function, and optimizer. The number of epochs used for the training of each model was different due to the differences in model overfitting, which is indicated by the ratio of training and validation loss gained during the model training. While the network architecture itself, network’s layers type, size, and connections between layers, directly influence the computational cost, the size of the trained model has influence in the inference due to the memory restrictions of the embedded hardware platforms. 

The new network we proposed in this paper, J-Net, had about 150 thousand trainable parameters, which was half of our implementation of PilotNet, that had about 350 thousand trainable parameters, and J-Net model had 280 times less parameters than the reimplementation of AlexNet, that had over 41 million trainable parameters, which means that we succeeded to deliver the least computationally demanding solution. The size of the J-Net trained model was four times smaller than the PilotNet model and about 250 times smaller than the AlexNet model. The smaller size of the network and the number of trainable parameters led to improvement of real-time performance in terms of latency reduction, and to the downsizing of the need for interfacing hardware in terms of computational power, cost, and space. 

Based on these results, we can say that our proposed network had less deep architecture than the other solutions we compared it with, a smaller number of trainable parameters, and, consequently, was the smaller trained model. This recommends the designed network for deployment on embedded automotive platforms. 

### 7.2. Performance Evaluation during Inference—Autonomous Driving

The verification of successful autonomous driving was done in the simulator on the representative track. During autonomous driving mode, the signal from the central camera mounted on the vehicle was continuously acquired and sent as an input to the trained machine learning model, which resulted with the control of the steering angle. Autonomous driving using all three models was recorded and given in the videos in [66,67,68] for AlexNet, PilotNet, and J-Net, respectively. As can be seen from the given videos, the J-Net fulfilled the requirement for autonomous driving in a predefined path, where the vehicle remained on the road for the full duration of the ride. The measurement of the performance was a successful drive on the representative track, the behavior that the vehicle does not get off the track during the ride, which implies that the better performing solution was the one where the vehicle was in the middle of the track for the full duration of the ride. The performance of the J-Net model was satisfactory. The qualitative performance evaluation of autonomous driving using implemented networks is given in Table 4.

One of the metrics for performance evaluation was observation of the vehicle behavior in curves, Table 4 second row. Among those three solutions, AlexNet performed best during the autonomous driving. Using AlexNet for autonomous driving, the vehicle was in the middle of the road most of the time, while during autonomous driving using PilotNet and J-Net, the vehicle was almost always in the middle of the road, but in some curves it came close to the edge. However, all three implementations of the autonomous driving succeeded to drive the vehicle on the road at all times, and did not go off the path. 

**Figure 16:**
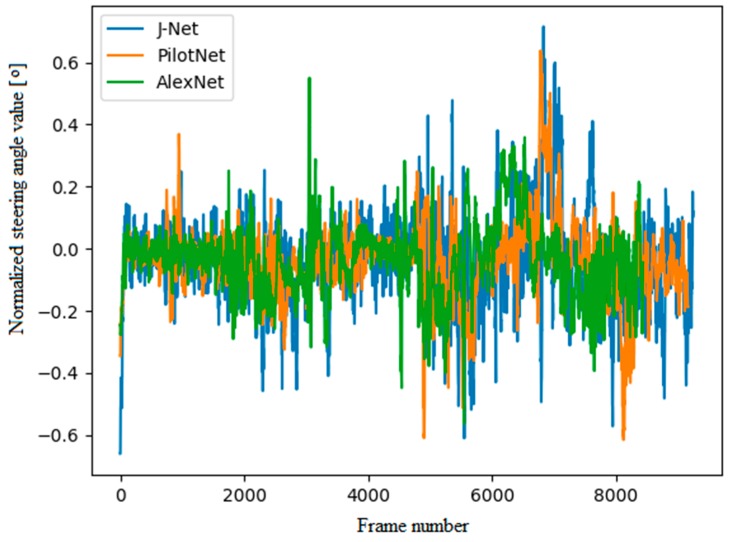
Steering angle predictions used for autonomous driving, presented as normalized absolute values of steering angle in degrees. J-Net has a blue plot, PilotNet orange, and AlexNet green.

In addition to observing autonomous driving on a representative track, the steering angle predictions used for autonomous driving were evaluated. As can be seen in Figure 16, the steering angle predictions for all three models were relatively similar. The graphical presentation of steering angle predictions used for real-time inference is given for one full lap of autonomous driving on the representative track. Positive and negative steering angle values represent the left and right angle of rotation. Since the representative track used for driving during inference was the same, and since the speed of the vehicle had been fixed due to simplicity, the steering angles in Figure 16 shows the steering angle predictions in similar frames. Steering angle predictions for the J-Net and PilotNet model were similar; however, the J-Net had a bit higher values, in both directions, left and right. The AlexNet model resulted with the mostly smooth steering predictions in the majority of the ride. However, in some points, it had extreme values, for example, at about 2500 frames there is a spike in the left direction, while the other two models did not have that sharp turn for that part of the road.

**Figure 17:**
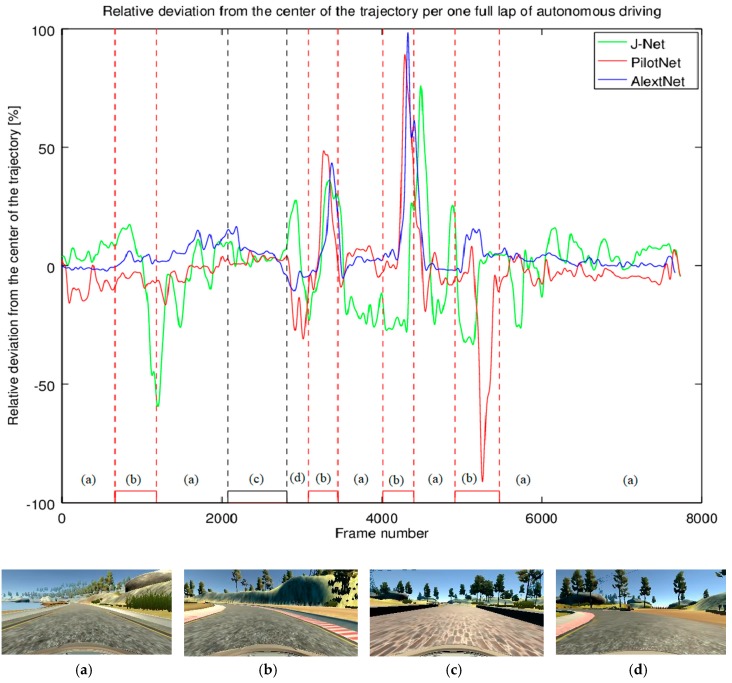
Relative deviation from the center of the trajectory per one full lap of autonomous driving. The four main characteristics of the trajectory are parts of the track defined with: (**a**) shoulders—regular, mostly straight road; (**b**) red and white stripe—mostly sharp curves; (**c**) a small wall—the bridge; (**d**) red and white stripe and dirt—a sharp curve.

As another measure of autonomous driving performance, we measured the impact of autonomous driving using each neural network on the trajectory. The relative deviation from the center of the trajectory per one full lap of autonomous driving is presented in Figure 17. The driving track characteristics may be seen as four main categories: a mostly straight part of the road rounded with shoulders, curves defined with red and white stripe, bridge, and parts of the road defined without any marks but with dust. As it can be seen from Figure 17, driving using all three models had similar patterns. The models show the car driving mostly without oscillations in straight parts of the road. However, in the curves, the deviation from the center of the trajectory was the biggest (e.g., after the bridge—the part of the diagram in Figure 17 marked as (c), there are three sharp curves—Figure 17d,b, while the third curve was the most challenging). Additionally, Figure 17 shows that all three networks had the deviations in this part, where AlexNet had the biggest deviation, and J-Net performed better than the other models. On the other hand, J-Net had more oscillations during the full lap, while AlexNet had the best performance, being closest to the center of the trajectory for most of the ride. 

**Figure 18:**
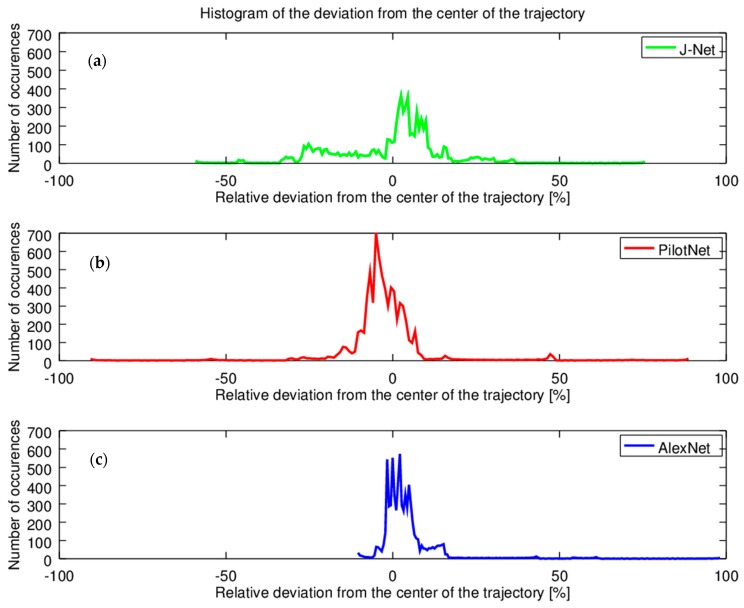
Histograms of deviation from the center of the trajectory per one full lap of autonomous driving. (**a**) J-Net; (**b**) Pilot Net; (**c**) AlexNet.

Statistical analysis of autonomous driving is also presented through the histograms. This analysis is significant for long term tests. In order to examine oscillations, the histogram of the relative deviations from the center of the trajectory per one full lap of autonomous driving is presented in Figure 18. Histogram of J-Net driving is presented in Figure 18a, where it was shown that the J-Net has the smallest deviation in the curves, while the oscillations for the center of the trajectory were the biggest. For the Pilot Net, Figure 18b, the oscillations were medium in comparison with using the two other networks for autonomous driving, but this model had significant oscillations in curves, both left and right, as it can be seen from Figure 18b, where for both directions sporadic occurrences had relative deviation from the center of the trajectory at almost 100%. Histogram of the relative deviation from the center of the trajectory shows that using AlexNet for autonomous driving, Figure 18c, had the most stable driving experience, with the smallest oscillations from the center of the trajectory. On the other hand, there was an occurrence of sporadic high deviation from the center of trajectory in one curve direction. However, this deviation was within the allowed limits, the car did not come out of the road, which was the criteria that we defined for successful autonomous driving.

Finally, all models performed well, successfully finishing the lap of autonomous driving with no significant deviation from the center of the trajectory. Differences between autonomous driving using different models were notable, but not large. 

Based on the computational complexity analysis, it was expected that J-Net would have the least latency and the highest frame rate among the three evaluated solutions. Quantitative performance evaluation verified this claim, as can be seen from Table 5. This evaluation was done on the PC platform explained in Section 6.2, which is a high-performance platform used for a simulator environment. The J-Net was able to successfully finish the task of autonomous driving on the representative track. As the track is a closed loop, we measured the number of successful consecutive laps for ten laps in total. All three models were able to successfully drive during the measured time. For the latency measurement, we calculated the time between two consecutive predictions. This number varies during the whole lap of autonomous driving, so for the latency, the mean value was used. The frames per second were calculated by counting the number of predictions per acquired frames in one second.

Observing the framerate measurements, using the J-Net model for the real-time inference was 30% faster than when using the AlexNet model on the high-performance platform used for the simulator environment. However, if we were using a scalar processor for the inference, the major differences would be expected (e.g., using J-Net over AlexNet would be 280 times faster). In the experiment where the simulator environment was used, the inferencing platform was a high-capacity computer with GPU that provided data parallelization. Hence, those results were for the particular application where the GPU was used. Here, since the neural network architectures were more different in their surface area than depth, the majority of operations were able to be done in parallel, so the difference in frame rate was the consequence of the sequencing in the algorithm execution, which was proportional to the network depth. 

The faithful demonstration of J-Net performance advantages is platform dependent. If we go to another extreme, when the operations are only done on scalar processors, it is expected that the execution rates would be much different, that is, comparable to the network capacity, the number of parameters. Real implementations of the J-Net model are intended for the embedded platforms, in which the degree of parallelization will be set so that the performance requirements in frame rate are met.

## 8. Conclusions

The development of high-performing computers able to perform training and inference for machine learning models leads to great advancement in novel approaches to known problems. However, the industrial application often requires machine learning solutions that can be deployed on computationally inexpensive and smaller memory demanding embedded platforms that have low cost and size. Deploying machine learning models on a low-performing hardware platform implies usage of an inexpensive models in terms of computational power and memory resources, which can be achieved by careful design of the neural network model architecture. In parallel with advancement in hardware development, in the development of novel processor units targeted for machine learning and, more precisely, deep learning applications, there is a trend in the design of light network architectures that can meet strict hardware requirements. 

The deep neural network presented in this paper is one possible solution for end-to-end learning for autonomous driving. The aim of our work was to achieve successful autonomous driving using a light deep neural network that is suitable for inference and deployment on an embedded automotive platform. Having this in mind, we designed and implemented J-Net, a deep convolutional neural network able to successfully perform the task of autonomous driving in a representative track, with the computational cost of using this network being the smallest among other known solutions that have been also explored in this paper. The main contribution of proposed work is the novel solution that is computationally effective due to relatively light architecture. The complexity of an algorithm is determined by the number of operations in one iteration, and our deep neural network has shown similar qualitative results gained with much fewer operations in comparison with other neural networks explored in this paper.

The possible limitation of J-Net could be the insufficient generalization for the more complex-use case scenarios. In addition to this, our model is trained using raw camera images and steering angle measurements per each frame, while the speed of the vehicle is taken as a constant due to the simplicity. This causes the limitation during autonomous driving regarding the speed since the constant speed is implied. However, it would be possible to train the J-Net to predict the speed of the vehicle. A similar approach to predicting steering angle can be used, which may lead to making simultaneous predictions for steering angle and speed based on the input camera image in real-time.

The future work will include the deployment of the presented network in an embedded automotive platform with limited hardware resources, low processor power, and low memory size. The possible final use cases for the presented end-to-end learning network are robot-cars in warehouses and delivery vehicles. Usage of light DNN solution, like the one presented in this paper, enables deployment on embedded automotive platforms with low-power hardware, low cost, and size, which is important for practical industrial applications. 

## Figures and Tables

**Table 1:** Dataset.

	Training	Validation	Total
Number of Samples	54,861	13,715	68,576
Percentage of Total Dataset	80%	20%	100%

**Table 2:** Layers and number of trainable parameters for J-Net, PilotNet, and AlexNet.

Layers and Parameters		AlexNet	PilotNet	J-Net
Convolutional		5	5	3
Flatten		1	1	1
Dense (fully-connected)		3	4	2
Total number of trainable parameters *		42,452,305	348,219	150,965
Operations	Multiplication	42.45 m	347.82 k	150.84 k
	Addition	42.45 m	347.82 k	150.84 k

* Total number of trainable parameters is calculated based on input image size. After lambda normalization and cropping, the size of input to the first convolutional layer is 65 × 320 × 3.

**Table 3:** Size of the trained models: J-Net, PilotNet, and AlexNet.

	AlexNet	PilotNet	J-Net
Number of epochs used for training	30	4	6
Size of the trained model	509.5 MB	4.2 MB	1.8 MB

**Table 4:** Qualitative performance evaluation of autonomous driving using AlexNet, PilotNet, and J-Net.

Autonomous Driving	AlexNet	PilotNet	J-Net
Autonomous driving on representative track	Successful	Successful	Successful
Handling the curves	Good	Medium	Medium/Good
Keeping to the trajectory center	Good	Medium	Medium
Driving on different surface textures	Good	Good	Good

**Table 5:** Quantitative performance evaluation of autonomous driving using AlexNet, PilotNet, and J-Net on a high-performance platform used for the simulator environment.

Autonomous Driving	AlexNet	PilotNet	J-Net
Number of successful laps *	10	10	10
Latency	26.0 ms	24.1 ms	23.8 ms
Frames per second	37 fps	42 fps	44 fps

* The autonomous driving is tested on 10 consecutive laps.
